# Loneliness and Social Isolation Among Centenarians and Near-Centenarians: Results From the Fordham Centenarian Study

**DOI:** 10.1093/geroni/igab046.416

**Published:** 2021-12-17

**Authors:** Daniele Zaccaria, Stefano Cavalli, Barbara Masotti, Daniela Jopp

**Affiliations:** 1 University of Applied Sciences and Arts of Southern Switzerland, Manno, Ticino, Switzerland; 2 University of Applied Sciences and arts of southern switzerland, Manno, Ticino, Switzerland; 3 University of Lausanne, Lausanne, Vaud, Switzerland

## Abstract

Although loneliness and social isolation are often discussed together, they are mainly examined separately. The few studies examining both concepts simultaneously focus usually on the wider category of older people (65+), with no or little attention to very old age. Our main aim was to investigate loneliness and social isolation in combination among near-centenarians and centenarians. Analyzing data from the Fordham Centenarian Study (N=94; MAge=99.2; range=95-107), we found no or very weak associations between loneliness and social isolation. Combining measures of loneliness (UCLA Loneliness scale) and social isolation (Lubben Scale) we built a typology with four different groups (Not lonely or isolated; Lonely and isolated; Lonely but not isolated; Isolated but not lonely). The factors that most strongly predicted the distribution among these four groups were gender, widowhood, education, and self-rated health. Findings highlight the importance of jointly studying both concepts to better understand social risks in very old age.

